# Strategy for the Generation of Engineered Bone Constructs Based on Umbilical Cord Mesenchymal Stromal Cells Expanded with Human Platelet Lysate

**DOI:** 10.1155/2019/7198215

**Published:** 2019-12-01

**Authors:** Ingrid Silva-Cote, Mónica Cruz-Barrera, Mariana Cañas-Arboleda, Luz Correa-Araujo, Leidi Méndez, Joanna Jagielska, Bernardo Camacho, Gustavo Salguero

**Affiliations:** Advanced Therapies Unit, Instituto Distrital de Ciencia Biotecnología e Innovación en Salud (IDCBIS), Bogotá, Colombia

## Abstract

Umbilical cord mesenchymal stromal cells (UC-MSC) are promising candidates for cell therapy due to their potent multilineage differentiation, enhanced self-renewal capacity, and immediate availability for clinical use. Clinical experience has demonstrated satisfactory biosafety profiles and feasibility of UC-MSC application in the allogeneic setting. However, the use of UC-MSC for bone regeneration has not been fully established. A major challenge in the generation of successful therapeutic strategies for bone engineering lies on the combination of highly functional proosteogenic MSC populations and bioactive matrix scaffolds. To address that, in this study we proposed a new approach for the generation of bone-like constructs based on UC-MSC expanded in human platelet lysate (hPL) and evaluated its potential to induce bone structures *in vivo*. In order to obtain UC-MSC for potential clinical use, we first assessed parameters such as the isolation method, growth supplementation, microbiological monitoring, and cryopreservation and performed full characterization of the cell product including phenotype, growth performance, tree-lineage differentiation, and gene expression. Finally, we evaluated bone-like constructs based on the combination of stimulated UC-MSC and collagen microbeads for *in vivo* bone formation. UC-MSC were successfully cultured from 100% of processed UC donors, and efficient cell derivation was observed at day 14 ± 3 by the explant method. UC-MSC maintained mesenchymal cell morphology, phenotype, high cell growth performance, and probed multipotent differentiation capacity. No striking variations between donors were recorded. As expected, UC-MSC showed tree-lineage differentiation and gene expression profiles similar to bone marrow- and adipose-derived MSC. Importantly, upon osteogenic and endothelial induction, UC-MSC displayed strong proangiogenic and bone formation features. The combination of hPL-expanded MSC and collagen microbeads led to bone/vessel formation following implantation into an immune competent mouse model. Collectively, we developed a high-performance UC-MSC-based cell manufacturing bioprocess that fulfills the requirements for human application and triggers the potency and effectivity of cell-engineered scaffolds for bone regeneration.

## 1. Introduction

Cell therapy strategies based on the use of mesenchymal stromal cells (MSC) have become an expanding tool for regenerative medicine. Increasing clinical evidence accumulated over the past years has demonstrated feasibility in the application of MSC-based therapies in terms of biosafety and therapeutic potential in a variety of pathologies associated with autoimmunity, chronic inflammation, and osteoarticular regeneration [1–3]. Along with the increasing set of data from preclinical and clinical research, there is an established consensus in regard to the criteria to identify MSC as well as the standardization procedures for cell manufacturing, improving reproducibility of cell products and comparability between clinical studies worldwide [4–7]. One of the major aspects that has an impact not only in therapeutic efficacy but also on the manufacturing process of human MSC therapy is the use of alternative sources for cell obtention, enhancing factors such as availability and feasibility of product scale-up for clinical use. An attractive source of MSC is the umbilical cord (UC), a by-product commonly discarded after pregnancy delivery. Based on preclinical and clinical evidence, UC-derived MSC exhibit similar biological and therapeutic properties when compared to classic cell sources such as bone marrow (BM) or adipose tissue (AD) [8–12]. UC-MSC display improved progenitor cell capacity and harbor strong differentiation potential towards mesenchymal lineages in a similar fashion as other cell sources [13–15]. Although therapeutic potential and biological mechanisms underlying tissue regeneration and repair of UC-MSC *in vivo* remain unclear, the introduction of UC-MSC as a therapeutic tool has opened new venues for clinical use in the allogeneic setting, taking into account that the use of MSC for clinical application has been mainly restricted to the autologous setting [4, 16]. Hence, establishing clinical-grade UC-MSC banks has become promising given the advantage of immediate availability of umbilical cord tissues for MSC-based therapy production, particularly in already established public cord blood bank facilities. As long as allogeneic use of MSC proves to be effective and safe, clinical-grade UC-MSC banks may provide unlimited access to cell therapies for regenerative therapy.

MSC have shown enormous potential in bone repair and healing in experimental and clinical settings [16]. Under appropriate culture conditions, MSC can differentiate into osteogenic lineages in a monolayer culture or in combination with three-dimensional (3D) scaffolds. Extensive evidence has shown that BM and AD-MSC are the main cell sources capable of inducing bone formation and trigger bone regeneration [17–19]. Despite experimental data showing bone healing and functional recovery in several injury models triggered by autologous adult BM or AD-MSC, engineered bone constructs using MSC from these sources still lack complete bone regeneration, in part due to the low numbers of viable and functional MSC used for the generation of tissue-engineered implants, which ultimately impacts their regeneration potential *in vivo* [20, 21]. This may be also explained by the fact that age and underlying pathological conditions of cell donors have a strong impact on stem cell products derived from adult tissues. MSC manipulation and expansion under current good manufacturing practice (GMP) protocols might lead to loss of proliferation and differentiation capacity towards committed bone progenitors [22]. For this reason, although potential of bone regeneration has not been fully demonstrated, UC-MSC represent an alternative source to trigger bone regeneration as they have shown bone differentiation properties *in vitro* and *in vivo* [10, 13]. Given the advantages of UC-MSC in terms of improved proliferation and multipotent lineage capacity, it is presumed a better *in vivo* performance and enhanced bone differentiation. Additionally, UC-MSC have shown potent immunomodulatory and proangiogenic properties which also make them excellent candidates for tissue engineering in the allogeneic setting. Importantly, GMP production of UC-MSC has not yet demonstrated to decrease multipotency and biological properties for clinical application [23, 24]. Importantly, diverse materials have also been used for development of scaffolds applied to bone regeneration such as bioactive ceramic, bioactive glass, and biological (collagen and hyaluronic acid) or synthetic (polylactic acid, polyglycolic acid, or polycaprolactone) polymers. The combination of these materials with UC-MSC aimed at triggering osteogenic differentiation has been previously reported for collagen I/collagen III [25], gelatin/bioactive glass [26], nanohydroxyapatite/chitosan/gelatin [27], and collagen/calcium phosphate scaffolds [28]. Overall, these studies showed a significant proosteogenic activity of UC-MSC when they were included in these constructs, suggesting feasibility of bone-like tissue construction with potential application in bone regeneration. Nevertheless, full understanding of the chemistry, size, and shape of the biomaterials and how they drive differentiation processes of UC-MSC remains a challenge.

In this study, we developed a new method for simple, homogeneous, and highly reproducible isolation and expansion of UC-MSC cell populations with increased cell yield. The development program for a therapeutic product for bone regeneration based on UC-MSC is here presented. Interestingly, we evaluated the capacity of UC-MSC to differentiate into bone *in vitro* and *in vivo* by using 3D constructs, pointing to tissue-engineered scaffolds as efficient tools for cell delivery directed to bone regeneration.

## 2. Materials and Methods

### 2.1. Umbilical Cord MSC Isolation and Establishment of Primary Cultures

UC were collected following screening of pregnant women at the hospital delivery board. Informed consent was reviewed and approved by the Ethical Committee of Secretaria Distrital de Salud de Bogotá, and it was signed by healthy donors prior to collection. Exclusion criteria for cord donors included sociodemographic variables such as age, nutrition status at pregnancy, and drug dependency as well as history of congenital anomalies, inborn metabolic or immune deficiencies, viral (varicella, papilloma virus, HIV, among others), bacterial, or parasite infections during pregnancy, eclampsia, and multiple pregnancy. Once cord tissue arrived to the center, cord blood was tested for HIV, HCV, HVB, Chagas disease, and syphilis positivity. In addition, cord tissues were subjected to examination in order to verify the presence of meconium, umbilical cord diameters below 1 cm, excess of blood in umbilical veins and artery, or cord below 15 cm. Admitted UC obtained from vaginal and cesarean deliveries of full-term newborns (*n* = 35) were sectioned (10 cm length) and immediately transferred to the processing facility in sterile containers loaded with phosphate-buffered saline (PBS) (Gibco, Life Technologies, Carlsbad, CA, USA) supplemented with 1% penicillin/streptomycin (P/S) 10000 U/10000 mg/mL (Gibco, Life Technologies, Carlsbad, CA, USA). In order to minimize hematopoietic cell contamination during cell derivation, we performed several washing steps with sterile PBS until flow through after washing was visibly clear. Three methods were compared for UC processing: explant processing (explant), UC enzymatic digestion (Col-tissue), and Wharton's Jelly (WJ) enzymatic digestion (Col-WJ). For explant processing, Wharton's Jelly (WJ) was separated from the UC tissue, transferred to 10 cm^2^ culture plates in DMEM supplemented with 10% human platelet lysate (hPL) plus 1% antibiotics and incubated at 37°C and 5% CO_2_. Cultured media were replaced every third day until cells reached 70% to 80% confluency. For UC enzymatic digestion, 3 cm sections were cut in small pieces, added to a solution containing 0.075% collagenase I (SigmaAldrich, St. Louis, MO, USA), and incubated for 60 min at 37°C in constant agitation. Cell suspension was passed through a 100 *μ*m mesh cell strainer, resuspended in hPL-supplemented DMEM, and seeded in a 6-well plate. Nonadherent cells were removed after 6 hours of culture. For Col-WJ, jelly was removed from the UC and digested in 0.075% collagenase I (SigmaAldrich, St. Louis, MO, USA) solution for 60 min at 37°C in constant agitation. Cells were subjected to separation in 100 *μ*m mesh and resuspended in DMEM supplemented with 10% hPL and 1% P/S. Cells were seeded in 6-well plates and maintained until confluency.

### 2.2. Microbiological Analysis

To assess the presence of microbial contamination at the collection and processing phases of UC, microbiological analyses were carried out in the PBS solution where the fragments were transported to the processing facilities, washing solution (PBS 1x, 1% P/S), and umbilical cord fragments and culture medium in passage 0 (P0) from 22 donors. Examination of samples and fluids for the presence of microorganisms was performed using blood culture bottles and thioglycolate broth. Thioglycolate broth was incubated for a period of 72 hours at 35°C and 5% CO_2_; the blood culture bottles were incubated in a standard atmosphere of 35°C for seven days for both aerobic and anaerobic germs using the microbial detection system BACT/ALERT® 3D (bioMérieux). Positive samples were stained with Gram and subcultured in blood agar and MacConkey and incubated for 72 hours at 35/36°C and 5% CO_2_; the isolated microorganisms were identified using the VITEK® 2 system (bioMérieux).

### 2.3. Extraction and Functional Characterization of Human Platelet Lysate (hPL)

hPL was obtained at the institutional blood bank from platelets of healthy blood donors (A+, B+, O+, and O-). Briefly, aliquots of 45 mL (3 donors) were frozen at −80°C and subjected to two freeze and thaw cycles to induce platelet lysis. Platelet lysates were then centrifuged at 4000g for 10 minutes, and supernatants were filtered through 0.2 *μ*m, aliquoted, and stored at −80°C until use. hPL from three donors was pooled and added to DMEM at a concentration of 10% for use in MSC culture. Human recombinant heparin was added at a final concentration of 16 IU/mL. In order to test the ability of hPL batches to support UC-MSC growth, cells from six donors were maintained in culture for 7 passages in the presence of DMEM supplemented with 10% hPL. Medium supplementation with Fetal Bovine Serum (FBS, 10%) was used as a control. Population doubling levels (PDL) for every passage were calculated using the formula *X* = [log10(NH) − log10(NI)]/log10(2), where NI is the inoculum number and NH is the number of cells harvested. The population doubling increase was also calculated by adding PDL level for every passage in order to obtain the cumulative population doubling (CPD). Furthermore, population doubling time was also calculated for every passage and expressed in hours per doubling. The content of cytokine and growth factors from different hPL preparations was determined by quantitative cytokine arrays using the commercial Luminex Human Cytokine 30-plex Assay (Invitrogen, Carlsbad, California, USA), following manufacturer instructions.

### 2.4. Assessment of Postcryopreservation Viability, Cell Recovery, and Long-Term Expansion

UC-MSC were harvested and resuspended in a final concentration of 1 × 10^6^ cells/mL. Cells were transferred to 1000 *μ*L precooled medium, containing DMEM and 10% dimethyl sulfoxide (DMSO) supplemented with either 30% of hPL (*n* = 22) or 30% FBS (*n* = 22). For each group, cells were then cryopreserved by either using a CryoMed controlled-rate freezer under a freezing rate of 1°C per minute or placing cryovials in precooled isopropanol racks (Mr. Frosty, Nalgene®) and transferring them to −80°C freezer for 24 h prior to final liquid nitrogen vapor storage. Cell samples were stored for one month until thawing. For viability and recovery assessment, UC-MSC were thawed and washed. Viable and dead cells were counted with a Neubauer chamber after staining with trypan blue (0.4%, Life Technologies). Cells were further seeded in 25cm^2^ tissue culture flasks (Corning, USA) in a density of 4.6 × 10^3^ cells/cm^2^ and seeded until confluency in order to obtain PDL, CPD, and PDT postthawing values. For long-term expansions, four MSC donors were kept in culture for up to 23 successive passages and PDL, CPD, and cumulative cell numbers were determined.

### 2.5. Immunophenotyping of UC-MSC

The expression of MSC-related cell surface antigens was assessed by flow cytometry using the membrane markers CD90 (APC), CD73 (PECy7), CD105 (PE), CD45 (APC/Cy7), CD34 (PerCP-Cy5.5), HLA-DR (Pacific Blue), and HLA-ABC (FITC). Cells were incubated for 30 min at 4°C, centrifuged at 300g for 6 min, and resuspended in 0.2 mL PBS. The procedure was performed in a FACSCanto II flow cytometer (Becton Dickinson, San Jose, USA), and data was analyzed with FlowJo vX.7.0 data analysis software package (Treestar, USA).

### 2.6. Differentiation of UC-MSC

The multilineage potential capacity of UC-MSC was examined by induction of cell differentiation using osteogenic, chondrogenic, and adipogenic differentiation media. Human adipose-derived and bone marrow-derived MSC were kindly donated by Dr. Jose Cardier at the Cell Therapy Unit, Instituto Venezolano de Investigaciones Científicas, Venezuela, and were included as positive controls for adipogenic, osteogenic, and chondrogenic differentiation. Adipogenic differentiation was carried out in 24-well plates seeding 5 × 10^4^ cells per well until 60% confluency and further incubating in adipogenic induction medium (StemPro Adipogenesis Differentiation Kit, Life Technologies) for 21 days. Cells were then fixed in 4% paraformaldehyde (SigmaAldrich, St. Louis, MO, USA) and stained with Oil Red O (SigmaAldrich, St. Louis, MO, USA). For osteogenic differentiation, cells were seeded as described above and exposed to osteogenic differentiation medium (StemPro Osteogenesis Differentiation Kit, Life Technologies). Calcium deposition was evidenced by Alizarin Red-S (SigmaAldrich, St. Louis, MO, USA) staining. Chondrogenic differentiation was assessed based on pellet formation; 1 × 10^6^ cells were centrifuged at 500g for 5 min at 10°C and resuspended in chondrogenic differentiation medium (StemPro Chondrogenesis Differentiation Kit, Life Technologies). Pellets were incubated at 37°C and 5% CO_2_ for 21 days. Following 21 days, pellets were washed 3 times with PBS, fixed in 4% paraformaldehyde solution for 24 hours, and embedded in paraffin. Four-micron sections (4 *μ*m) were prepared and stained with Masson trichrome to evaluate the presence of collagen (blue) and chondroid cells.

### 2.7. Quantitative RT-PCR

Total differentiated and undifferentiated UC, BM, and AD-MSC RNA were isolated by a PureLink RNA Mini Kit (Thermo Fisher Scientific, Waltham, Massachusetts, USA) according to the manufacturer protocol. RNA concentration and quality were assessed in a NanoDrop-1000 instrument (Thermo Scientific NanoDrop™ 2000/2000c). Complementary DNA was prepared by reverse transcription of total RNA with SuperScript™ IV First-Strand cDNA Synthesis Reaction (Invitrogen, San Diego, CA, US), followed by qRT-PCR in a 7500 Fast Real-Time PCR System (Applied Biosystems, California, USA) using TaqMan gene expression assays (Applied Biosystems, California, USA). Analyzed genes included bone-specific marker genes, SPP1 (secreted phosphoprotein 1, Hs00959010_m1) and BGLAP (osteocalcin, Hs01587814-g1), adipose-specific genes FABP4 (Hs01086177_m1) and PPARG (Hs01115513_m1), cartilage-specific genes, COMP (cartilage oligomeric matrix protein, Hs00164359_m1) and FMOD (fibromodulin, Hs00157619_m1), and housekeeping genes HPRT (Hs02800695_m1) and *β*-actin (Hs01060665_g1). PCR efficiency for each gene was determined based on the calibration curve using the formula *E* = 10^[‐1/slope]^ − 1. Relative expression was subsequently calculated using 2-^*ΔΔ*CT^ method.

### 2.8. Generation of Bone Scaffolds

UC-MSC were seeded and conditioned to differentiate towards osteogenic or endothelial lineages in T25 culture flasks. UC-MSC were incubated with osteogenic (StemPro Osteogenesis Differentiation Kit, Life Technologies) or endothelial induction media (medium 200, Life Technologies) for 72 hours. Osteogenic and endothelial-induced cells were then harvested, combined in a 3 : 1 ratio (75000 osteogenic and 25000 endothelial), and resuspended in 1 mL of culture medium containing 100 *μ*L of atelocollagen microbeads (Koken Co., Ltd.) thus generating constructs. Cell-collagen constructs were incubated at 37°C and 5% CO_2_ for 24 hours. Next, culture medium was removed and constructs were mixed with 50 *μ*L of human platelet-rich plasma (PRP) and 10 *μ*L of 25% CaCl_2_ and thrombin (ratio 1 : 1) until clot formation was observed. Constructs cultured with DMEM supplemented with 10% hPL were also prepared as controls. Constructs were either directly implanted for testing *in vivo* bone formation or subsequently cultured to evaluate bone differentiation *in vitro* by osteogenic stimulation for additional 14 days. Constructs were finally fixed in 4% paraformaldehyde, embedded in paraffin, and analyzed by conventional histology and immunohistochemistry.

### 2.9. Bone Formation *In Vivo*

Cell-collagen constructs were subcutaneously implanted in 6-week-old female C57BL/6 mice. Briefly, mice (*n* = 6) were anaesthetized by intraperitoneal injection of 0.1 mL/kg xylazine and 0.12 mL/kg ketamine, and cell constructs were placed aseptically on the dorsal subcutaneous area. Mice were maintained with standard chow diet and water ad libitum. Twelve weeks post implantation, mice were sacrificed by cervical dislocation followed by extraction of cell constructs. Constructs were further fixed in 4% paraformaldehyde and embedded in paraffin for histological analyses.

### 2.10. Histological Analysis

Paraffin blocks from *in vivo* and *in vitro* constructs were sectioned (4 *μ*m) and stained with hematoxylin and eosin (H&E) and Masson trichrome for collagens (blue). Osteogenesis was evaluated by Alizarin Red-S (SigmaAldrich, St. Louis, MO, USA) staining. For immunohistochemistry analyses, sections were blocked for 1 hour at room temperature (RT) with 5% BSA and 5% FBS in PBS 1x and then incubated with primary antibodies against Col I (SC25974, St. 28 Cruz), overnight at 4°C. The next day, samples were washed three times with PBS and incubated with secondary antibodies conjugated with HRP (R&D Systems, MN, Canada) for 1 hour at RT and finally washed with PBS and mounted for microscope evaluation.

### 2.11. Statistical Analysis

In order to determine statistic significances, we used Student *t*-tests and ANOVA for parametric data and the Kruskal-Wallis test for nonparametric data. The level of significance was considered when the *p* value was below 0.05. Statistical analysis was carried out using the GraphPad Prism version 6.0 software (La Jolla, CA, USA).

## 3. Results

### 3.1. Isolation, Culture, and Immunophenotype of Umbilical Cord-Derived MSC

**Figure 1:**
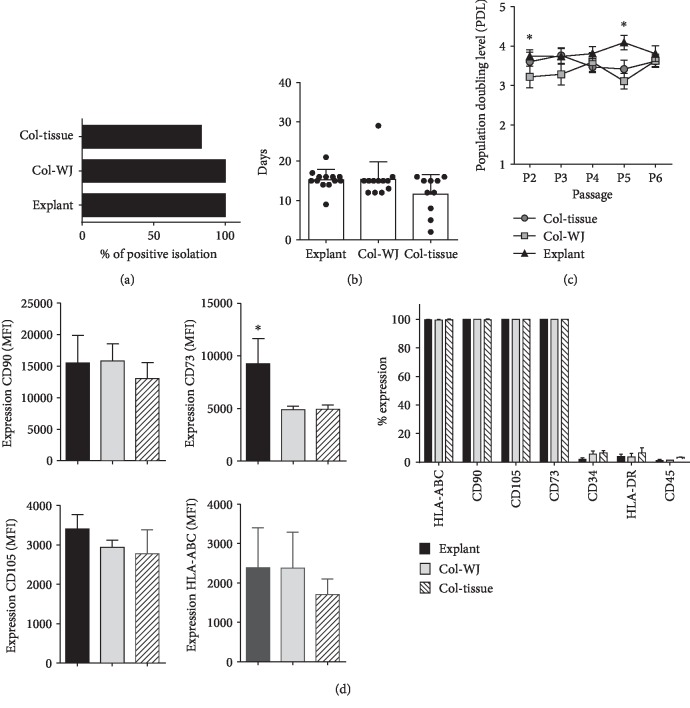
Initial characterization of UC-MSC according to three isolation methodologies. (a) Percentage of positive umbilical cords (UC) processed by each isolation method (*n* = 10‐12 donors per group). (b) Time of cell derivation (P0 to P1) for every isolation method (*n* = 10‐12 donors per group). (c) Population doubling time measured in early cell passages (P2 to P6) per isolation method (*n* = 10 donors per group). (d) Flow cytometry analyses of MSC identity markers (CD90, CD73, CD105, HLA-AB, HLA-DR, CD45, and CD34) as shown in total frequency. Level of expression for CD90, CD73, CD105, and HLA-Ab is presented as Median Fluorescence Intensity (MFI) (*n* = 4 donors per group). ∗ indicates *p* < 0.05, as evaluated by ANOVA.

Several approaches have been described for isolation of MSC. Here, we compared three methods: collagenase-based enzymatic digestion of whole umbilical cord tissue (Col-tissue), Wharton's Jelly matrix isolation and collagenase digestion (Col-WJ), and WJ matrix explant (explant) in a total of 35 cord donors. We assessed efficiency, purity, and cell growth performance. When assessing the efficiency of the three isolation methods, we found higher cell derivation rates of MSC in explant (*n* = 13) and Col-WJ (*n* = 12), compared to Col-tissue isolations (83.3%, *n* = 10) (Figure 1(a)). We observed early cell sprouting and adhesion to plastic in Col-tissue-isolated MSC at day 11.6 ± 4.9, compared with derivation times for explant and Col-WJ (*p* > 0.05) (Figure 1(b)). However, cultures obtained by the Col-tissue method showed heterogeneous cell populations, one fibroblast-like morphology population and the other small rounded population. In contrast, cultures obtained from Col-WJ and explant isolates displayed characteristic fibroblast-like MSC phenotype and absence of other cell types at culture initiation (data not shown). Cells derived from primary cultures (passage 0) were further expanded, and population doubling levels (PDL) were calculated for all cell cultures up to passage 6. We observed consistent PDL values between 3 and 4 in all cultures, regardless the isolation protocol used initially (Figure 1(c)). Notably, MSC isolated by explant showed consistent and homogeneous PDL levels as compared to the other methodologies for cell isolation. In order to characterize MSC, we evaluated the expression of MSC identity markers at passage 1 by flow cytometry. The expression percentage of CD105, CD90, CD73, and MHC class I reached 99% (Figure 1(d)); cells also displayed negative expression (less than 5%) of hematopoietic lineage markers CD34, HLA-DR, and CD45. Next, we compared the mean fluorescence intensity (MFI) for each marker on cells isolated by Col-tissue, Col-WJ, and explant protocols. Explant MSC showed a higher expression of CD73 and CD105 as compared to Col-WJ and Col-tissue (Figure 1(d)) indicating a stronger preservation of MSC phenotype after explant isolation. Importantly, explant methodology showed significantly enhanced expression of CD73, suggesting a higher enrichment of progenitor cells within isolated MSC populations. Together, these results probed explant methodology to enrich populations harboring enhanced MSC phenotype in early isolated MSC.

### 3.2. Microbiological Monitoring of Cord Tissues and Derived UC-MSC

Since microbiological contamination is a critical factor for quality compliance and batch release of cell-based medicinal products, we wanted to screen microbial contamination by monitoring very early stages of tissue collection and cell processing. We first tested contamination in samples of UCs collected from cesarean and vaginal births at different time points throughout the whole processing chain including transport from hospital to the cell processing facility. To this end, we monitored tissue samples, washing solutions, and culture medium at P0. Samples were seeded into aerobic and anaerobic blood culture bottles and thioglycolate broth for further microbiological evaluation.

**Figure 2:**
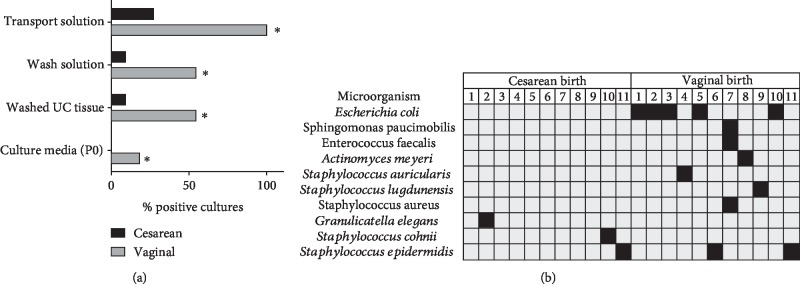
Microbiological monitoring of umbilical cord tissue, transport and washing solutions, and culture media used during isolation and initial culture of UC-MSC. (a) Percentage of contaminated samples comparing cesarean and vaginal deliveries (*n* = 11 donors per group). (b) Microorganisms identified in contaminated donors (filled squares) collected in cesarean and vaginal deliveries. ∗ indicates *p* values < 0.05 when comparing cesarean vs. vaginal delivery as evaluated by Chi-squared and Fisher exact tests.

Vaginal, fecal, and skin microbiota microorganisms were detected in 100% of vaginal delivery samples of transport solution (*n* = 11, Figure 2(a)). In contrast, only 30% of samples obtained from cesarean delivery (*n* = 11) were found positive. As expected, after several washing steps in antibiotic solution, the overall level of microbiological contamination and in particular washing solutions and tissues was drastically reduced in both from vaginal birth and cesarean samples. Taking into account the previous results, we characterized microorganisms isolated from positive samples and observed the presence of *E. coli* and *Staphylococcus* sp. bacterial strains in most of the screened cords (Figure 2(b)). Almost all bacterial isolates resulting from microbiological evaluation were sensitive for wide spectrum antibiotics such as cephalothin, gentamicin, and vancomycin (Table 1). Thus, higher microbiological contamination in UC obtained from vaginal deliveries greatly increases the risk of further contaminated cell cultures. Nevertheless, appropriate manipulation and eventual use of prophylactic antibiotic cocktails such as first- and second-generation cephalosporin (cephalothin, cefoxitin), gentamicin, tobramycin, or vancomycin in transport media will prevent contamination, especially in those cords obtained from vaginal deliveries.

### 3.3. Xeno-Free Growth Media Support Expansion of UC-MSC

**Figure 3:**
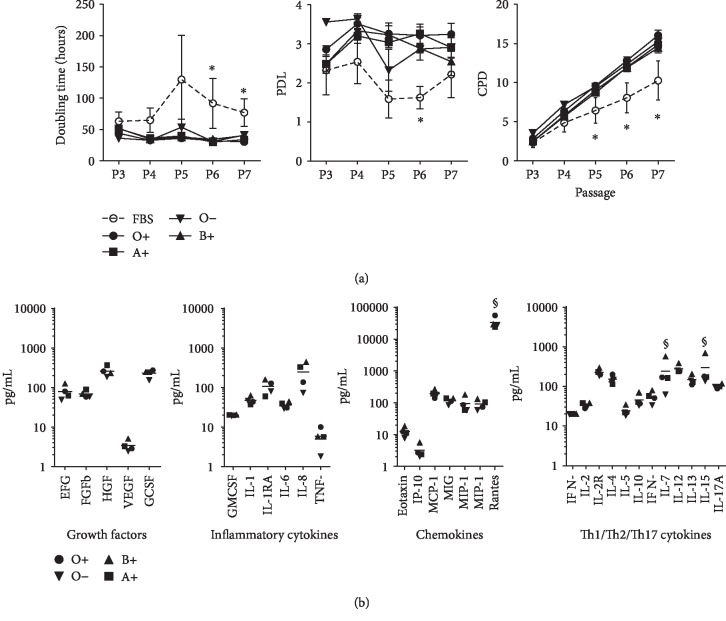
Impact of the use of human platelet lysate (hPL) as a medium supplement for cell culture of UC-MSC. (a) Proliferation kinetics of UC-MSC as measured by population doubling time (hours), population doubling levels (PDL), and cumulative population doublings (CPD) per passage. Pooled batches of hPL from different blood groups were evaluated and compared with Fetal Bovine Serum (FBS) supplement (*n* = 4 donors per group). (b) Comparison of human cytokine levels (pg/mL) measured in different hPL batches obtained from blood donors (*n* = 3 per blood group). ∗ indicates *p* < 0.05 when comparing FBS vs. hPL as evaluated by ANOVA and Tukey's multiple comparison. § indicates *p* < 0.05 as tested by ANOVA.

An essential aspect to be addressed during the manufacturing process of cell-based products is the use of growth factor supplements that meet the criteria for human use. We standardized the production of human platelet lysate (hPL) derived from platelet bags for cell culture. In order to assess comparability between batches of hPL, we pooled up to three platelet bags per blood group (A+, B+, O-, and O+) and subjected them to freeze-thaw cycles, filtration, and storage for further use as a supplement in MSC cultures. To evaluate the impact of donor variation on cell growth, MSC (*n* = 4 donors) were seeded at passage 3 in culture media containing 10% of different hPL pools. We used 10% FBS as the control. No significant differences were found in the population doubling level (PDL), population doubling time (PDT), and cumulative population doubling (CPD) values in cultured MSC exposed to hPL obtained from A+, B+, O-, and O+ groups (Figure 3(a)). Furthermore, we found stable cell growth kinetics throughout evaluated passages, with averaged PDL of 3.2. In contrast, cells cultured in FBS-supplemented media exhibited significantly lower PDL values. Finally, higher and more stable cell proliferation rates of MSC exposed to hPL-supplemented culture medium resulted in higher CPD values when compared with MSC cultured in FBS media (*p* < 0.05). In order to assess the presence of several growth factors contained in hPL, we compared growth factors, inflammatory cytokines, chemokines, and Th1/Th2/Th17 cytokine concentrations among different batches and blood groups. We observed similar concentrations in the majority of the cytokines, chemokines, and growth factors evaluated for hPL from all batches derived from A+, O-, and O+ donors. Only IL-15, IL-7, and RANTES showed significant variation among blood groups and hPL pools (*p* < 0.05, Figure 3(b)). Overall, hPL pools prepared from different blood groups showed homogeneous content of growth factors and cytokines and demonstrated feasibility to support the expansion of MSC.

### 3.4. Effect of MSC Cryopreservation in Viability, Recovery, and Growth Performance

**Figure 4:**
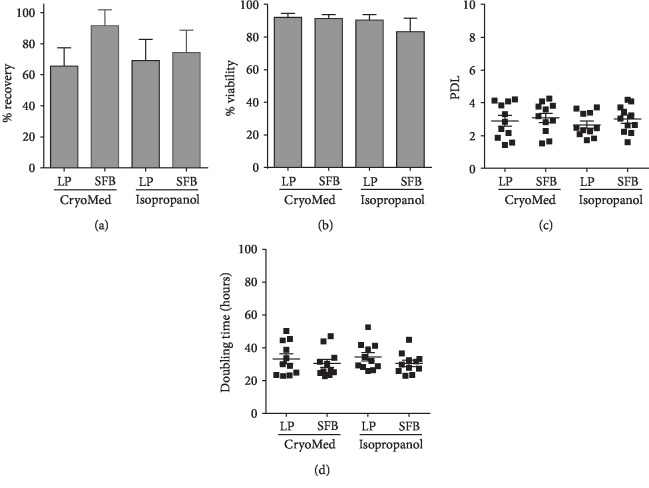
Viability, recovery, and growth rate of UC-MSC evaluated after cryopreservation. (a) Cell viability after thawing represented as percentage of alive cells obtained (*n* = 7 donors per group). (b) Cell recovery after thawing (*n* = 7 donors per group). (c) Population doubling levels (PDL) and (d) cell doubling time assessed in UC-MSC cultures (*n* = 12 per group) obtained after different cryopreservation methods.

Given the easy access and availability of cord tissue in our public umbilical cord blood bank and the possibility to immediately provide cell therapy products from third party allogeneic donors for clinical application, we sought to stablish an allogeneic master cell bank of isolated UC-MSC. We first assessed whether the cryopreservation process might impact MSC viability and growth performance. Here, we tested different cryopreservation strategies on MSC (*n* = 44) at passage 1: freezing medium containing 10% FBS or 10% hPL as well as the use of two methodologies for cell cryopreservation: precooled isopropanol containers (Nalgene® Mr. Frosty) at minus 80°C for 48 h followed by transfer to liquid nitrogen or CryoMed Controlled-Rate Freezers followed by direct transfer to a liquid nitrogen tank. Two weeks post cryopreservation, cells were thawed and cell recovery and viability were tested by trypan blue exclusion. For MSC cryopreserved in a CryoMed device, we observed cell recovery rates from 67 to 81% when media were supplemented with FBS or hPL, respectively (*p* > 0.05) (Figure 4(a)). Similarly, when isopropanol containers were used, we observed cell recovery rates from 60.5% for hPL-supplemented freezing media. Accordingly, viability after thawing was around 90 ± 6% for all conditions except for those cells cryopreserved in FBS-supplemented freezing medium frozen in isopropanol containers, where we found decreased viability levels (Figure 4(b)). In order to determine the direct impact of cryopreservation protocols on cell growth, thawed cells were cultured until confluence (around day 4) and PDL and PDT values were determined. We did not find any significant differences in PDL values when comparing hPL-freezing media and media supplemented with FBS for both freezing methodologies (*p* > 0.05) (Figure 4(c)). As expected, PDT levels averaged 30 to 33 hours (*p* > 0.05, Figure 4(d)). Taking all together, we found cryopreservation in CryoMed devices to be superior in terms of cell recovery and viability. Furthermore, the use of hPL as a supplement for freezing media was probed to maintain cell viability and growth capacity of MSC when cryopreserved under xeno-free conditions.

**Figure 5:**
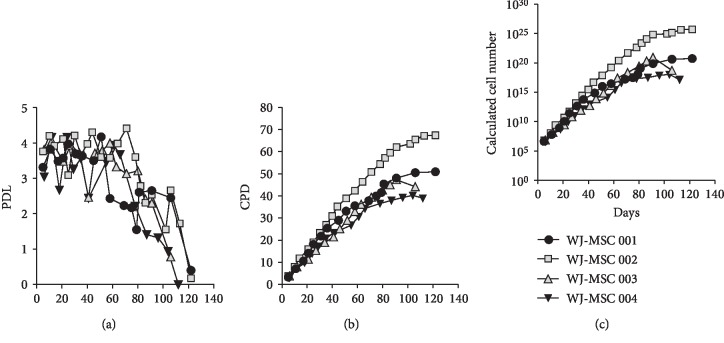
Long-term proliferation kinetics and potential cell yield of UC-MSC cultures. (a) Population doubling levels (PDL) and (b) cumulative population doublings (CPD) of UC-MSC cultures from four different umbilical cord (UC) donors maintained for up to 23 passages. (c) Theoretical total cell counts calculated according to cell growth kinetics observed in the evaluated UC donors.

Finally, we evaluated the long-term impact of cryopreservation and cell manipulation on MSC fitness. We cultured MSC (*n* = 4) for up to 120 days (around passage 20) and evaluated the kinetics of cell growth. In all four donors, we observed PDL values between 3 and 4 up to day 40 (passage 12) (Figure 5(a)). Interestingly, from day 63 (passage 15), we observed sustained decrease of PDL, reaching levels below 1 at day 110 (passage 19). Along with this observation, we found dramatic changes in MSC morphology, displaying a larger cytoplasm size at late passages associated with strong reduction of cell density. Consequently, MSC cultured beyond 65 days displayed a significant reduction of CPD values (Figure 5(b)). Thus, PDL and CPD values at some extent can predict long-term performance of MSC growth. Interestingly, by using MSC cultures reaching CPD values below 40, we could potentially obtain a cell yield ranging 10^10^, which in turns will allow multidose and multipatient preparation for MSC-based cell therapies (Figure 5(c)).

### 3.5. Differentiation Assessment of MSC Derived from UC

**Figure 6:**
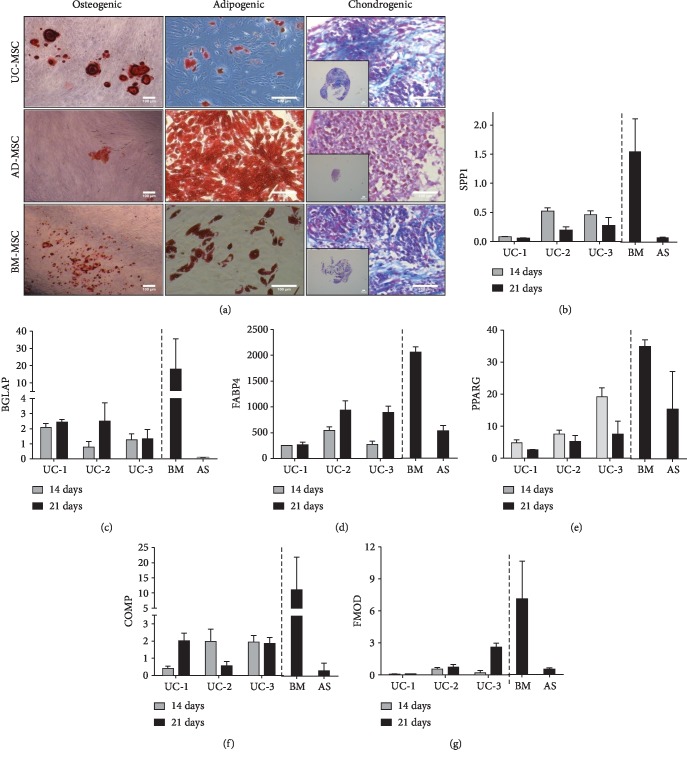
Mesenchymal lineage differentiation of UC-MSC. (a) Cultured UC-MSC (passages 5-6) were induced towards osteogenic and adipogenic lineages, and differentiation was verified by Alizarin Red and Oil Red staining, respectively. For chondrogenic differentiation, UC-MSC (*n* = 3 donors) pellets were incubated in chondrogenic induction media and micromasses were evaluated by Masson's trichrome staining. Bone marrow- (BM-) and adipose tissue- (AD-) derived MSC were used as controls for differentiation. Embedded images show representative size of cartilage-like pellets (bar indicates 100 *μ*m). Relative gene expression (qRT-PCR analysis) of adipocyte-, osteocyte-, and chondrocyte-related genes in UC-MSC (*n* = 3 donors) after 14 and 21 days of differentiation. Gene expression levels of (b) SPP1, (c) BGLAP, (d) FABP4, (e) PPAR*γ*, (f) COMP and (g) FMOD are presented as fold increase relative to the housekeeping gene. Gene expression was also evaluated in BM and AD-derived MSC and used as controls.

We next tested whether there is a correlation between UC-MSC gene expression and differentiation towards osteo-, adipo-, and chondrogenic lineages (Figure 6(a)). We evaluated messenger RNA (mRNA) expression of osteogenic (SPP1, BGLAP), adipogenic (FABP4, PPAR*γ*), and chondrogenic (COMP and FMOD) genes by quantitative RT-PCR. Regarding proosteogenic genes, we detected the expression of SPP1 in UC-MSC subjected to osteogenic differentiation, with a peak at day 14. Compared to controls, BM-MSC expressed the highest level of SPP1, in contrast to AD-MSC, where we found no SPP1 expression (Figure 6(b)). Similarly, BGLAP expression was observed already at day 14 and was maintained up to day 21 in treated UC-MSC (Figure 6(c)). However, BGLAP expression in BM-MSC seemed to be superior as compared to UC-MSC and AD-MSC. In the case of adipogenic-related gene expression after adipocyte induction, we detected a high expression of FABP4 in UC-MSC on day 14 which significantly increased on day 21 (Figure 6(d)). Similar expression was also found in BM-MSC and AD-MSC after 21 days of differentiation treatment. The expression of adipogenic PPAR*γ* mRNA was also detected in UC-MSC at days 14 and 21 after differentiation (Figure 6(e)), although higher levels of gene expression were found in BM-MSC and in AD-MSC at day 21. Finally, we evaluated the expression of chondrogenic-related genes FMOD and COMP in MSC pellets following chondrogenic induction protocol. We detected FMOD and COMP expression on days 14 and 21 in differentiated pellets from UC-MSC (Figures 6(f) and 6(g)) also found in BM-MSC and AD-MSC. Taken together, these results confirmed that upon specific stimulation, UC-MSC can differentiate into osteogenic, adipogenic, and chondrogenic lineages and display a similar pattern of gene expression as observed for AD-MSC and BM-MSC. Thus, UC-MSC harbor multilineage potential and therefore might be used for regenerative purposes in tissue engineering.

### 3.6. UC-MSC Support Formation of Bone-Like Structures in 3D Scaffolds *In Vivo*

**Figure 7:**
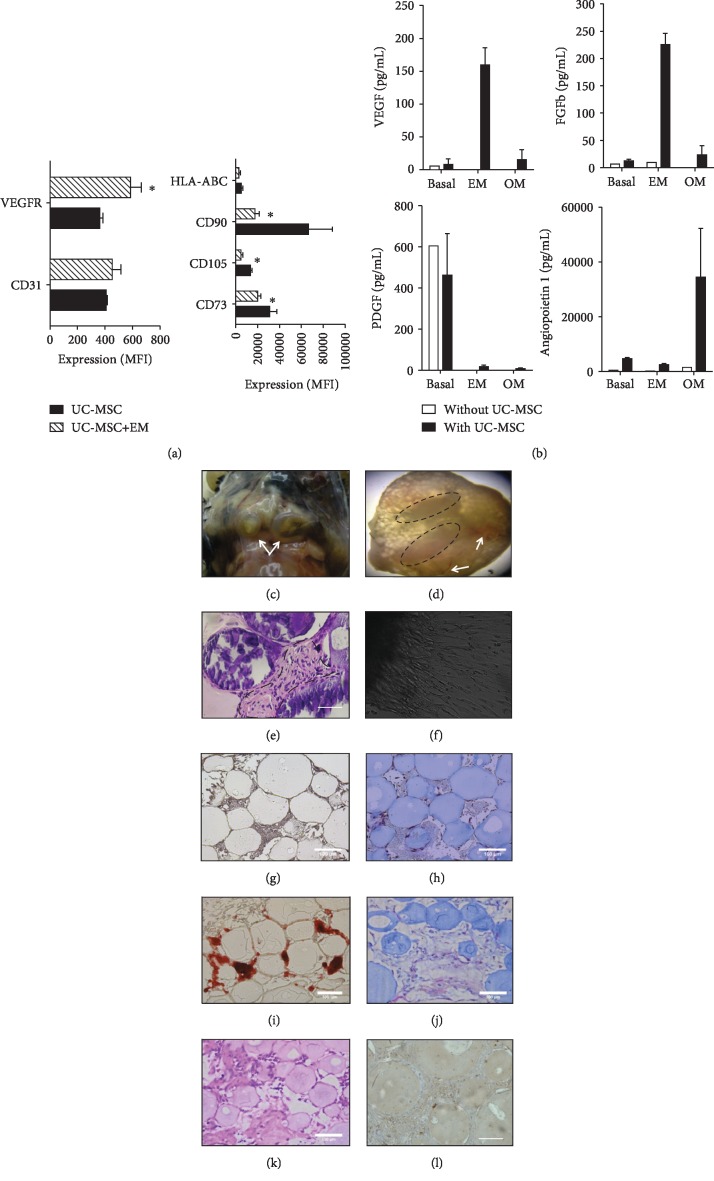
*In vivo* formation of bone-like scaffolds based on UC-MSC. (a) Expression of endothelial and MSC markers in cells treated with endothelial induction (EM) or growth media. (b) Concentration of growth and angiogenic factors in EM, OM-treated UC-MSC, or nontreated (basal) controls. (c) Bone-like tissue formation at 12 weeks after transplantation of UC-MSC-microbeads in mice. (d) Mineralization zones are evident at the upper part (circles) and angiogenesis (arrows) in bone-like tissue formed from UC-MSC. (e) Hematoxylin and eosin staining evidence the presence of osteoid cells of immature appearance (dotted line) in bone-like tissue formed from UC-MSC scaffolds. (f) Cell migration outside explants of *in vivo*-formed bone-like tissue after 48 h of culture. Representative micrographs of bone constructs in growth medium (g) and differentiation medium (h) stained for Alizarin Red after 14 days of culture. Representative micrographs of bone constructs without (i) and with UC-MSC (j) stained with Masson's trichrome cultured for 14 days. Micrographs of hematoxylin and eosin (k) and collagen (l) staining of 14-day cultured scaffolds containing UC-MSC. Differences between expressions of different markers were compared. ∗ indicates *p* < 0.05. Bars indicate 100 *μ*m.

Considering the potential of UC-MSC to differentiate to osteogenic lineages, we finally sought to test whether UC-MSC cultured on 3D-collagen scaffolds could enhance their capacity to form bone-like structures. We first evaluated whether MSC were able to induce bone-like structures in a previously established model of scaffolds based on collagen microbeads [29]. To that end, we preconditioned two groups of UC-MSC (passage 5) for 3 days in the presence of proosteogenic medium (UC-MSC/OM) or proendothelial differentiation medium (UC-MSC/EM). After conditioning treatment, UC-MSC/OM and UC-MSC/EM were cultured on collagen microbeads in a cell ratio of 3 : 1 (UC-MSC/OM : UC-MSC/EM) and further embedded in human plasma clot. We first evaluated the effect of endothelial or osteogenic treatments in MSC preconditioned with OM and EM media. Cells were analyzed for endothelial (CD31 and VEGFR) and MSC cell markers by flow cytometry. UC-MSC exposed to EM did not show significant changes in CD31 expression; however, they showed an enhanced expression of Flk1 marker (VEGF receptor, *p* < 0.05, Figure 7(a)). Also, UC-MSC/EM showed significantly lower expression levels of the MSC identity markers CD90, CD73, and CD105 (*p* < 0.05, Figure 7(a)) than UC-MSC alone. On the other hand, cells exposed to OM treatment maintained high levels of MSC markers without changes in the expression of VEGFR or CD31 (data not shown). We also evaluated the release of proangiogenic cytokines and growth factors from UC-MSC exposed to EM or OM. We observed a significant increase of VEGF and basic FGF in supernatants of UC-MSC induced with EM when compared to basal or OM (Figure 7(b), *p* < 0.05). Angiopoietin 1 release was also induced upon stimulation with osteogenic media (*p* < 0.05 vs. basal and EM). Interestingly, we did not observe release of PDGF in the medium supernatant of EM or OM-treated cells. Thus, conditioning of UC-MSC with proangiogenic or osteogenic signals induces the expression of key factors such as VEGF, FGF, and angiopoietin that could potentially enhance the viability of scaffold *in vivo*. Next, we implanted UC-MSC-collagen microbeads immediately after OM/EM preconditioning into C57BL6 mice and later removed them at week 12. At the time of harvest, implants (Figure 7(c)) were extracted and fixed in 4% PFA, embedded in paraffin, and stained with Alizarin Red, hematoxylin and eosin, and Masson trichrome. Interestingly, we observed development of blood vessel-like structures and darken structures (Figure 7(d)), suggesting that 3D-culture configuration promotes a more intense calcium deposition on the extracellular matrix in preconditioned UC-MSC. Histological analyses confirmed the presence of osteoid cells of immature appearance in the implanted tissues (Figure 7(e)). When sections of *in vivo*-derived bone scaffolds were placed in culture, we could observe outgrowth of cells with mesenchymal phenotype, indicating maintenance of UC-MSC cell growth *in vivo* (Figure 7(f)). We also monitored bone formation in bone constructs maintained *in vitro*. Following 14 days of culture, we assessed Ca deposition (Alizarin Red) and collagen matrix formation (Masson trichrome staining and hematoxylin and eosin, Figures 7(i)–7(k)), indicating immature bone tissue formation, arrays of fine and coarse collagen fibers, and immature osteocytes adjacent to the collagen matrix formed. Control constructs stained negative for both Alizarin Red and Masson's trichrome (Figures 7(g) and 7(h)). We further confirmed the presence of collagen fibers by immunohistochemistry (Figure 7(l)). Taken together, UC-MSC loaded in collagen microbeads were able to induce bone-like structures and maintained vitality *in vitro* and *in vivo*. Based on these data, conditioning of UC-MSC induced a functional proangiogenic and osteogenic phenotype which led to marked vascularization and bone formation *in vivo*.

## 4. Discussion

In this report, we developed a strategy to generate a cell therapy product based on UC-MSC with potential use in bone engineering for clinical applications. We aimed to introduce a reproducible protocol for MSC isolation, expansion, and banking in accordance with critical standards for cell production under good manufacturing practices, including the establishment of critical parameters for quality control and the use of xeno-free reagents for cell expansion. Importantly, we assessed the feasibility of the construction of bone-like scaffolds based on collagen microbeads combined with UC-MSC and tested its potential for bone formation *in vitro* and *in vivo*.

Regeneration of bone injuries has been the target for stem cell-based therapies over the past decade, in particular, in those conditions associated with major bone defects, loss of bone substance, or delayed fracture union. In spite of the fact that MSC application showed relative clinical success in improving osteonecrosis [30–32], mandibular and bony defects [33–37] or fracture remodeling, full recovery of the bone structure and function remains a challenge for tissue engineering approaches on the bone. These previous clinical experiences have been mostly based on the use of adult bone marrow, adipose tissue, or dental pulp-MSC, delivered alone or in combination with a variety of chemical or biological scaffolds, and showed a wide range of clinical outcomes from symptom alleviation to full bone mineralization. However, clinical data still lacks enough power to draw conclusions about the efficacy of MSC for bone regeneration, mostly due to small and heterogeneous cohorts, diversity of cell delivery routes, and variations in the source and characteristics of the cell product [38, 39]. In this scenario, the use of UC-MSC becomes attractive for the generation of clinical-grade bone constructs. Our strategy takes advantage of the availability of umbilical cord tissue collected in a public cord blood bank facility, in order to generate standard cell banks for further clinical use. Importantly, previous reports have addressed the opportunity of developing such MSC banks for “off-the-shell” applications, based on relevant clinical data demonstrating a strong safety profile in particular on the allogeneic clinical setting [24]. Here, we obtained reproducible numbers of MSC from the Wharton Jelly tissue when a technique for cell derivation based on tissue explant was applied. Importantly, cell purity, immunophenotype, morphology, and cell growth kinetics demonstrated highly homogenous and reproducible cell products. These observations agree with the great potential of MSC from UC to generate cell-based therapeutics for a large scale as well as for personalized medical applications.

We demonstrated the capacity of UC-MSC to differentiate to osteogenic lineages *in vitro* when cultured as monolayer or seeded on 3D-culture structures, as well as *in vivo*. Even though BM and AD-derived MSC have been widely used as the cell source for bone tissue engineering [17–19], UC-MSC have also demonstrated potential of osteogenesis [40]. Considering the fact that BM or AD-MSC may contain relatively less mesenchymal progenitors [20, 21] and these cells progressively lose the capacity to proliferate and differentiate into osteoblasts during cell culture manipulation and expansion [22], UC-MSC becomes an ideal source of cells for culture and clinical-scaling as they might provide substantial increase of osteogenic progenitor numbers at the initial harvest. We could confirm osteogenic differentiation of UC-MSC at 21 days in a similar fashion as observed in the BM-MSC and identified a similar gene expression profile of key factors associated with osteogenesis *in vitro*. Interestingly, we also observed enhanced chondrocyte formation in UC-MSC as compared to BM or AD-derived cells, displaying remarkable chondrocyte-like differentiation and increased proteoglycan and collagen production. These observations support the notion that UC-MSC might be enriched with more osteogenic and chondrogenic progenitor cell populations and therefore might constitute a very efficient source of bone-inducer cells with regenerative properties. Furthermore, we were also able to show improved proangiogenic activity of the construct together with the absence of inflammatory response from the host. Vascularization of the bone scaffold is critical for the generation of viable and functional constructs [41–43]. Here, not only the differentiation properties of the implanted cell but also the release of key angiogenic and growth factors are relevant to preserve the minimal conditions for bone induction of the engineered construct *in vivo* [44–46]. The strategy used in this report revealed that UC-MSC not only acquire a bone-like phenotype but also, when induced towards vascular phenotype, become sensitized to VEGF signaling hence triggering the release of growth and proangiogenic factors that favorably impacts construct vascularization. Thus, upon adequate stimuli, UC-MSC-containing scaffolds showed bone induction and proangiogenic properties leading to a highly viable, biologically active allogeneic construct that can ensure its viability post implant, minimizing tissue necrosis and adequately inducing bone repair.

In order to generate efficient bone constructs for tissue engineering-based therapy, MSC-derived products must satisfy several critical criteria for cell production including robust capacity to expand *in vitro*, reproducibility in cell yields, and proven potency of cell product here shown as differentiation capacity prior to administration [6, 24]. In the process of developing cell-based medicinal products, high regulatory and quality standards involving good manufacturing practices come into question to further the transfer to the clinical setting. In the case of MSC, protocols for cell manufacturing starting from ex vivo cell derivation up to large-scale expansion have shown to be feasible and cost-effective using basic cell culture strategies in appropriate cell manipulation facilities. The process described in this study was developed to obtain a rapid and consistent method of homogeneous cell populations for later generation of bone-like constructs. Consistent with previous reports [9, 40], we observed that MSC could easily be isolated from WJ under controlled conditions allowing efficient cell isolation using explant or tissue digestion with collagenase from either whole cord tissue or only WJ. However, the use of collagenase as a previous step for cell isolation has shown reduced reproducibility and efficiency, in particular, when scaling up to GMP production [47]. On the other hand, tissue explant has been extensively described as suitable for cell derivation and manufacturing for clinical use [48–50]. In our study, derivation times (from P0 to P1) averaged 13.5 days after explant, allowing high cellular yields—around 10^10^ cells—after 30 to 40 cell duplications. Thus, cell expansion protocols based on WJ explant followed by rapid expansion support the generation of reliable cell products with adequate cell fitness. Importantly, previous data from adult adipose and bone marrow-derived MSC showed early signs of culture-induced senescence early after 30 population doublings [51]. Furthermore, the increase of cumulative population doublings in BM or AD-derived MSC has been associated with a high risk of cell transformation [52]. Our data supports the possibility of generating a large number of cells within an acceptable cell doubling range; however, only after prolonged passaging (CPD > 60) do we start to observe replicative senescence. This data suggests that neonatal MSC are able to maintain longer proliferation capacity in culture, hence extending its therapeutic window while displaying a safety profile for *in vivo* applications. Interestingly, UC-MSC have not been reported to induce teratoma *in vivo* [15, 24] but showed a gene expression profile similar to the observed in embryonic stem cells (ESC), suggesting improved cell stemness. UC-MSC are in addition obtained from discarded birth delivery material and has unlimited availability, and their collection, processing, preservation, and clinical use do not imply any ethical issues [53]. Thus, compared to BM or AD sources, UC-MSC display important advantages for effective use as cell therapy. Other critical factors to be addressed during cell production relate to the use of a growth factor source to supplement culture media in order to ensure high cell performance while guaranteeing an adequate biosafety profile for human applications. We established a reproducible protocol to generate human use-compliant culture media based on human platelet lysate. The use of hPL as a medium supplement for MSC production has increasingly gained popularity due to the feasibility and reproducibility of production and the strong evidence supporting consistent cell expansion under GMP [54]. Here, we were able to confirm that the addition of hPL to cell culture media supported stable MSC growth and maintained osteogenic and proangiogenic features. Importantly, this effect was significantly superior to the standard cell culture supplement FBS. Furthermore, we even probed feasibility of the use of hPL as a supplement for cryopreservation of MSC. In line with these observations, we confirmed no difference in the beneficial effect of hPL on MSC performance when different blood group donors were employed for medium supplement preparation. This is supported by the fact that hPL pools derived from four different blood groups did not show significant differences in the concentration of cytokines, chemokines, and growth factor content in hPL among batches.

In summary, here, we have addressed key factors to develop a high-performance cell manufacturing bioprocess based on UC-MSC that ultimately not only ensure biosafety requirements for human application but also improve potency and effectivity of bone-like constructs for tissue engineering-based therapies. In the clinical scenario, the cell-engineered construct generated here can act as a biological inductor of bone regeneration in patients with subacute segmented bone defects associated with trauma or chronic defects associated with atrophic or congenital pseudoarthrosis. In these conditions, the cell construct can supply additional bone-differentiated cell components and three-dimensional support improving the formation of bone callus. In addition, due to the secretion of growth and proangiogenic factors here observed, we can expect *in vivo* induction of neoangiogenesis and increased blood supply, thereby reducing tissue necrosis and triggering cell migration, survival, and bone consolidation. Ultimately, the application of such cell-engineered bone-like construct might support a full bone regeneration, avoiding amputation and other severe sequels in those complicated bone defects. The use of UC-MSC for clinical applications continues its expansion for regenerative medicine purposes. However, despite the increasing amount of preclinical and clinical data favoring the use of MSC as therapy for the correction of bone injuries or defects, there are still several questions to be addressed in order to overcome the limitations of these approaches for extensive clinical use. It is widely accepted the safety profile of UC-MSC as medicinal agents in the clinical setting, but therapeutic efficacy continues to be debated. In this regard, deep understanding on the identification of specific progenitor cell populations prone to bone differentiation and the molecular signals driving osteogenesis from mesenchymal stages should still be intensively carried out in UC-MSC preparations. On this basis, the use of new biocompatible materials mimicking bone substructures and allowing 3D incorporation of cellular compounds to trigger full integration of the artificial bone construct within damaged host tissue is warranted. The incorporation of strategies for the selection and expansion of specific osteogenic cell populations, the induction and maintenance of osteoinductive signals, and their combination with proper 3D-scaffolds within the manufacturing pipeline will improve the applicability of this novel therapeutics for bone regeneration.

## Figures and Tables

**Table 1:** Minimum Inhibitory Concentration (MIC) of antibiotics tested on bacterial isolates after microbiological screening of umbilical cords.

Microorganism		Minimum Inhibitory Concentration (MIC) in *μ*g/mL								
		Ampicillin		Cephalothin		Gentamicin		Vancomycin		
		MIC	SI	MIC	SI	MIC	SI	MIC	SI
*Escherichia coli*	Gram-negative bacilli	≤2	S	4	S	≤1	S	—	—
*Sphingomonas paucimobilis*		—	—	4	S	≤1	S	—	—
*Staphylococcus cohnii*	Coagulase-negative staphylococci (CoNS)	—	—	—	—	≤0.5	S	1	S
*Staphylococcus epidermidis*		—	—	—	—	4	S	≤0.5	S
*Staphylococcus auricularis*		—	—	—	—	≤0.5	S	1	S
*Staphylococcus lugdunensis*		—	—	—	—	4	S	≤0.5	S
*Staphylococcus saccharolyticus*		—	—	—	—	1	S	1	S
*Staphylococcus aureus*	Coagulase-positive staphylococci	—	—	—	—	≤0.5	S	1	S
*Enterococcus faecalis*	*Enterococcus*	≤2	S	—	—	—	—	≤0.5	S

Abbreviations: SI = susceptibility interpretation; S = sensitive; I = intermediate; R = resistant.

## Data Availability

The data used to support the findings of this study are available upon request.
